# How spatial accessibility to colonoscopy affects diagnostic adherences and adverse intestinal outcomes among the patients with positive preliminary screening findings

**DOI:** 10.1002/cam4.3054

**Published:** 2020-04-21

**Authors:** Weiyi Chen, WangJian Zhang, Huazhang Liu, Yingru Liang, Qin Zhou, Yan Li, Jing Gu

**Affiliations:** ^1^ Department of Medical Statistics School of Public Health Sun Yat‐sen University Guangzhou People’s Republic of China; ^2^ Department of Environmental Health Sciences University at Albany State University of New York Rensselaer NY USA; ^3^ Department of Noncommunicable Chronic Disease Control and Prevention Guangzhou Center for Disease Control and Prevention Guangzhou People’s Republic of China; ^4^ Sun Yat‐sen Global Health Institute Institute of State Governance Sun Yat‐sen University Guangzhou People’s Republic of China

**Keywords:** colorectal cancer, epidemiology and prevention, risk assessment, screening

## Abstract

**Background:**

Colonoscopy is an important procedure for early colorectal cancer (CRC) detection, however, patients with positive preliminary screening results in China may not seek for colonoscopy to confirm the diagnosis. We evaluated the spatial accessibility of colonoscopy among the residents with positive preliminary screening results in Guangzhou, China, and investigated how colonoscopy accessibility was associated with the population adherence and adverse intestinal outcomes.

**Methods:**

This study was based on the Guangzhou community‐based CRC screening program. Spatial accessibility was measured using three metrics including travel time from home to nearest colonoscopy hospital, physician‐to‐population ratio (PPR) and accessibility indicator estimated with enhanced two‐step floating catchment area method (E2SFCA). We used Cox regression and logistic regression to assess the association of colonoscopy accessibility with population adherence and adverse intestinal outcomes, respectively.

**Results:**

A total of 34 606 people were identified with positive preliminary screening findings. Central areas were reported with higher E2SFCA scores, higher PPR and less travel time. The model adjusting for potential individual level confounders found that PPR > 50 (Hazard Ratio (HR) = 1.88, 95% Confidence Interval (CI): 1.79‐1.97) and higher scores of E2SFCA (HR = 3.78, 95% CI: 2.07‐6.92) were associated with increased adherence, although estimates were not significant in the model adjusting for both individual and district‐level confounders. For adverse intestinal outcomes, the final multilevel logistic model suggested a lower risk of intestinal lesions among the residents in areas with PPR > 50 (Odds Ratio (OR) = 0.49, 95% CI: 0.24‐0.99) and higher scores of E2SFCA (OR = 0.20, 95% CI: 0.05‐0.82).

**Conclusion:**

Significant inequality of colonoscopy accessibility was observed across Guangzhou. The increased incidence of intestinal lesions was associated with spatial inequalities of medical resources. Policies against the spatial inequality in medical resources should be developed.

## Graphical abstract

While previous studies suggested that colonoscopy nonadherence was an important issue mitigating the efficiency of colonoscopy promotion programs in China, this study investigated the spatial inequity of colonoscopy accessibility in a capital city in South China as well as its relationship with colonoscopy adherence and adverse intestinal outcomes.

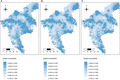

## 1 INTRODUCTION

According to the World Cancer Statistics Report of 2018, colorectal cancer (CRC) is among the top three most common cancers worldwide. It is also one of the leading causes of cancer deaths, accounting for 9.2% of the total cancer deaths worldwide in 2018.^1^ In China, the age standardized incidence rate (ASIR) of CRC was 17.52 per 100 000 people in 2014.^2^ Urban areas were generally reported with higher rate than the national average. In some Chinese cities, the ASIR of CRC was higher than 20 per 100 000 people.^3^ In Guangzhou, the ASIR OF CRC was 24.47 per 100 000 people in 2014.^4^

Colonoscopy is an important procedure for early CRC detection, therefore, is critical for minimizing the adverse impact related to CRC.^5^, ^6^, ^7^ However, patients with positive preliminary screening results in China may not seek for colonoscopy to confirm the diagnosis, which mitigates the impact of early diagnosis and treatment programs on promoting the population wellness. Previous studies suggested that low colonoscopy adherence (colonoscopy adherence was defined hereafter as high CRC risk population receiving colonoscopy) is a common problem in China with the percent of people with positive preliminary screening results receiving colonoscopy in urban areas ranging from 7.0% to 47.0% across China.^8^, ^9^, ^10^, ^11^, ^12^ Therefore, prevention strategies targeted on potential causes of colonoscopy, no adherence are urgently needed with the aim of reducing the economic and health burden related to CRC.

Previous studies have investigated the potential association of low colonoscopy adherence with multiple individual factors including age, occupation, income,^13^, ^14^, ^15^ awareness and knowledge on CRC, fear of colonoscopy, and health insurance coverage.^16^, ^17^, ^18^ Programs targeting on these factors have significantly improved the colonoscopy rate across China through health education, short messages notification, and lowering the cost of colonoscopy.^19^, ^20^ However, in some areas, the impact of these programs was limited and significant gaps remain in our understanding whether factors other than individual factors should be considered in developing CRC prevention strategies.

According to previous studies,^21^, ^22^, ^23^ spatial accessibility of health services (colonoscopy in this study) was also an important predictor for the population adherence. Spatial accessibility represents the relationship between the supply capacity of existing services and the needs of residents (defined as the availability), and the relationship between the location of the service and the residential address (defined as the accessibility).^21^ Generally, studies used physician‐to‐population ratio (PPR) to represent the availability, and travel distance/time to evaluate the accessibility.^22^, ^24^ Based on previous findings, improving the spatial accessibility of colonoscopy by shortening the traveling distance between hospitals providing colonoscopy and patients’ home was associated with a higher colonoscopy adherence^25^ and a higher identification rate of CRC.^26^ In contrast, longer travel distance could be associated with increased prevalence of metastatic disease, inferior cancer‐specific, as well as higher readmissions among the CRC patients.^27^, ^28^, ^29^ However, most of these studies focused on the impact of the accessibility of colonoscopy. Little is known about how the accessibility and availability of colonoscopy simultaneously affect the colonoscopy adherence and related health outcomes, particularly in south China.

Based on these knowledge gaps, this study was aimed to evaluate the spatial accessibility (accessibility and availability) of colonoscopy among the people with positive preliminary screening results in Guangzhou, a capital city in South China, and to explore its association with the colonoscopy adherence and adverse intestinal outcomes.

## 2 METHODS

### 2.1 Study design

This study was conducted based on the Guangzhou community‐based CRC screening program. This program was initialized in 2015 and targeted on residents aged 50‐74 years based on the Optimization of Sequential Screening Scheme which included two steps: (a) the program using a high‐risk factor questionnaire (HRFQ) and two immunochemical fecal occult blood tests (iFOBTs) to identify the patients with positive preliminary screening findings, and (b) colonoscopy to confirm the diagnosis of CRC among these patients. Specifically, once a patient with positive preliminary screening findings received a colonoscopy examination, all relevant information (including the date and results of the examination) on this examination would be upload to the CRC screening system and matched with other information for the same patient according to a unique ID.

This retrospective cohort study was conducted in all the 11 administrative districts of Guangzhou, covering 14.5 million residents (2018).^30^ The participants were recruited between January 1, 2015 and December 31, 2016, and were followed up through December 31, 2017.

### 2.2 Study population

Among the residents with positive preliminary screening results in the CRC screening program in Guangzhou, we included those who: (a) lived in Guangzhou for more than 6 months; (b) were 50‐74 years old; (c) reported a complete home address (the address for 95.5% cases in urban districts and 56.1% cases in rural districts was detailed to the number of apartment. However, the house/apartment number was not available for the rest of cases who lived in the countryside of the urban and rural districts, in which case the address of village was used instead); (d) had a clear diagnosis determined by doctors and uploaded to the screening system; (e) signed an informed consent to participate in the CRC screening program, and was aware that the data would be used for analysis. We excluded those reported with a colonoscopy contraindication. The study was approved by the ethics committee of School of Public Health, Sun Yat‐sen University.

### 2.3 Data collection

**FIGURE 1:**
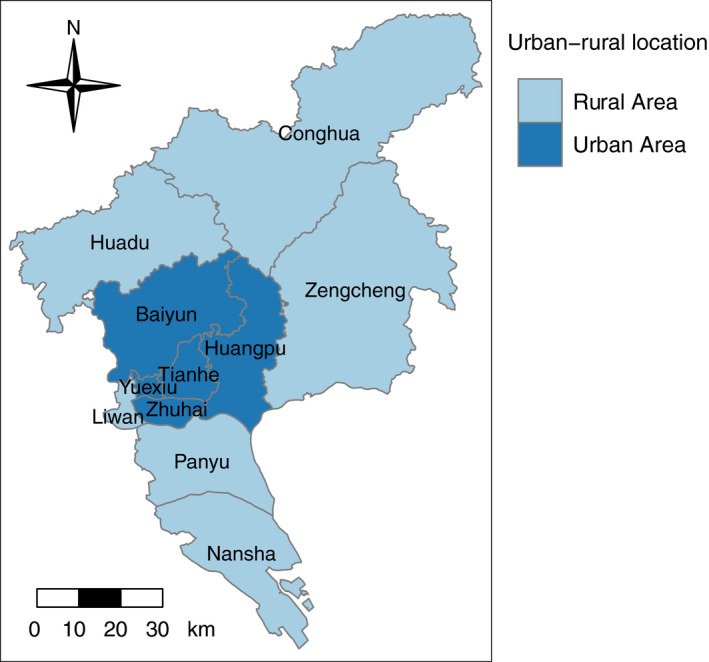
The Urban‐rural pattern of Guangzhou

We obtained the predictor data at both the individual and district levels. The individual‐level information of participants was collected from the CRC screening database, including age (using the 50‐54 group as the reference), gender (using female as the reference), marital status (using married as the reference), educational level (using primary school or lower as the reference), occupation (current or before retirement, using government or public institution as the reference), health insurance (using medical insurance for urban workers as the reference), home address, preliminary screening results (using only HRFQ‐positive as the reference), and colonoscopy examination (time and results). We grouped the health insurance into four categories as (a) medical insurance for urban workers, (b) medical insurance for urban residents, (c) free medical service, and (d) other. Free medical service refers to a social security system that the Chinese government provides free medical and preventive services to national staffs. The district‐level data were collected from the Guangzhou Center for Disease Control and Prevention (CDC), including discount on the cost of colonoscopy (using no discount as the reference) and rural‐urban location (using Urban area as the reference). The discount for colonoscopy varied across administrative districts. In this study, we grouped the districts in the study area into three categories as (a) districts providing no discount, (b) districts providing a discount of 100‐300 (RMB) per colonoscopy examination and (c) districts providing free colonoscopy examinations. We classified 11 districts in Guangzhou into urban and rural areas as suggested by the Guangzhou Municipal Health Commission on the CRC program. Although there might be socioeconomic disparities between people living in the same district, medical services and benefit (such as the discount on the cost of colonoscopy exam) were generally consistent across different groups, as administrated by the policy of the district authorities. Urban areas were generally located in the central of the city as was shown in Figure 1. Covariates in the current study including result of preliminary screening and urban‐rural location were selected based on previous studies.^31^, ^32^

Hospital information was acquired from the Guangzhou Health Information Center, which included the list of hospitals that could provide colonoscopy services, the addresses of hospitals, and the number of personnel (doctors) in gastroenterology in each hospital. We totally identified 68 hospitals in Guangzhou providing colonoscopy service, among which three could provide the service in two hospital campuses. Therefore, in this study, we have 71 hospital locations for colonoscopy service.

### 2.4 Geographic measures

All the colonoscopy hospitals and participants were geocoded by address based on the application programming interface (API) of Baidu Maps. Since public transportation was the major method for the residents to reach colonoscopy hospitals in China, we estimated the public transportation time from each participant's home to the nearest colonoscopy hospital to represent the accessibility of colonoscopy. However, when the distance from the residential address to the nearest colonoscopy hospital was short while no public transportation was available, we used walking time instead. In this study, we defined multiple subzones as 0‐10, 10‐30, and 30‐60 minutes of travel time. The study area, Guangzhou City, contains 11 districts as displayed in Figure 1. For each district, we used a physician‐to‐population ratio (PPR) to estimate the average availability of relevant medical resources for the residents in that district, which was calculated as doctors in gastroenterology per thousand people with positive preliminary screening findings of each administrative district.

Spatial accessibility to colonoscopy hospitals were estimated for each participant using the enhanced two‐step floating catchment area method (E2SFCA) proposed by Luo and Qi (2009),^33^ which is a combined indicator of accessibility and availability. The E2SFCA method subdivides a catchment into several subzones and applies different weights to different subzone, which takes the geographical variation within the catchment into consideration^33^, ^34^, ^35^ and makes the results more interpretable and easier to apply. This method has been widely used in previous studies.^34^, ^36^, ^37^ The method includes two steps as following:

First step: we first identified the zone that could be reached within 60 minutes from the colonoscopy hospitals using public transportation, and then computed three travel time subzones by 0‐10, 10‐30, and 30‐60 minutes (subzones 1‐3, *D_r_*) according to other studies conducted in the China.^34^, ^38^ Finally, for each colonoscopy hospital *j*, all the population locations *k* in each subzone (*D_r_*) were searched, and then the weighted PPR *R_j_* of each colonoscopy hospital was calculated:

(1) $$\begin{array}{cc} R_{j}= & \frac{S_{j}}{\sum_{k\in \left\{d_{\mathrm{kj}}\in D_{r}\right\}}P_{k}W_{r}}\\ = & \frac{S_{j}}{\sum_{k\in \left\{d_{\mathrm{kj}}\in D_{1}\right\}}P_{k}W_{1}+\sum_{k\in \left\{d_{\mathrm{kj}}\in D_{2}\right\}}P_{k}W_{2}+\sum_{k\in \left\{d_{\mathrm{kj}}\in D_{3}\right\}}P_{k}W_{3}} \end{array}$$

*P_k_* was the population of each population location *k* with its centroid falling within the catchment *j* (*d_kj_* ∈ *D_r_*). In this study, we used detailed residential address for each participant. *S_j_* represented the supply capability in catchment *j*. *W_r_* was the distance weight for the subzones, which was used to reflect the influence of distance attenuation on accessibility. *W_r_* was calculated by Gaussian function $\left(f\left(d_{\mathrm{ij}}\right)=e^{-d_{\mathrm{ij}}^{2}/\beta }\right)$ . Based on previous sensitivity analysis and other studies conducted in China,^34^, ^36^, ^38^, ^39^ we set β to 440, and the weights of the three subzones to 0.9448, 0.4029, and 0.0100, respectively.

Second step: For each population location *i*, we searched for all colonoscopy hospitals that could be reached within 60 minutes of public transportation, and then summed up the *R_j_* at these settlements from the first step:

(2) $$A_{i}^{F}=\sum_{j\in \left\{d_{\mathrm{ij}}\in D_{r}\right\}}R_{j}W_{r}=\sum_{j\in \left\{d_{\mathrm{ij}}\in D_{1}\right\}}R_{j}W_{1}+\sum_{j\in \left\{d_{\mathrm{ij}}\in D_{2}\right\}}R_{j}W_{2}+\sum_{j\in \left\{d_{\mathrm{ij}}\in D_{3}\right\}}R_{j}W_{3}$$

$A_{i}^{F}$ represented the spatial accessibility of participants at population locations *i* to the colonoscopy hospitals. In this study, it represented the distance‐weighted ratio of number of medical staffs per people with positive preliminary screening results. $A_{j}^{F}$ was a relative indicator rather than the absolute value, therefore, it should be applied to the relative comparison.^33^, ^36^, ^40^ An E2SFCA score of 0 represented that a participant was unable to obtain services at any colonoscopy hospital within 60 minutes.

We performed spatial interpolation (Inverse distance interpolation) in Guangzhou based on the public transportation time and spatial accessibility of each population location, then generated the maps based on the results of spatial interpolation.

### 2.5 Outcome

Colonoscopy adherence was defined as receiving colonoscopy at the colonoscopy hospital during the follow‐up period. Intestinal lesions included ulcer, ulcerative colitis/Crohn's disease, chronic inflammation, adenoma/polyp and malignant tumors which were identified with colonoscopy. CRC included the diagnosis of malignant tumors by colonoscopy.

### 2.6 Statistical analyses

First, we fitted univariate Cox regression models to estimate hazard ratio (HR) of colonoscopy nonadherence during the follow‐up period, using individual and district‐level variables listed in Table 1 as independent variables. We then included variables with *P* < .05 from the univariate analysis into a forward stepwise multivariate Cox regression based on Akaike information criterion (AIC). Second, we developed univariate and multivariate models adjusting for the individual‐level background confounders selected by the multivariate Cox regression in the previous step, using the travel time, PPR, and E2SFCA scores as independent variables to estimate the *HR* of colonoscopy nonadherence. Finally, we developed a multilevel Cox regression model with individuals (level 1) nested in districts, adjusting for the individual and district‐level (discount on the cost of colonoscopy and rural‐urban location) background confounders.

**TABLE 1:** Background characteristics among the people with positive preliminary screening results (34 606), people underwent colonoscopy during follow‐up (8026), and people with intestinal lesions (4255)

	People with positive preliminary results			People underwent colonoscopy			People with intestinal lesions		
	n	%	Adherence %	n	%	Lesion %	n	%	Cancer %
*Individual‐level variables*									
Age (y)									
50‐54	3517	10.2	26.9	946	11.8	43.9	415	9.8	3.1
55‐59	5051	14.6	25.3	1276	15.9	48.7	622	14.6	4.7
60‐64	7941	22.9	23.6	1873	23.3	52.6	985	23.1	4.3
65‐74	18 097	52.3	21.7	3931	49.0	56.8	2233	52.5	6.6
Gender									
Male	13 565	39.2	25.0	3388	42.2	61.7	2090	49.1	6.5
Female	21 041	60.8	22.0	4638	57.8	46.7	2165	50.9	4.4
Educational level									
Primary school or lower	10 672	30.8	19.0	2027	25.3	55.7	1129	26.5	6.2
Secondary school	19 355	55.9	24.1	4659	58.0	52.6	2451	57.6	5.3
College or higher	4579	13.2	29.3	1340	16.7	50.4	675	15.9	4.6
Occupation									
Government or public institution	9175	26.5	23.7	2177	27.1	55.1	1199	28.2	5.8
Enterprise	3487	10.1	29.1	1013	12.6	51.9	526	12.4	3.2
Peasant	5701	16.5	19.9	1133	14.1	53.4	605	14.2	5.6
Unemployed	4245	12.3	22.0	936	11.7	53.1	497	11.7	5.0
Other	11 998	34.7	23.1	2767	34.5	51.6	1428	33.6	6.0
Marital status									
Married	31 136	90.0	23.7	7369	91.8	53.0	3908	91.8	5.6
Other	3470	10.0	18.9	657	8.2	52.8	347	8.2	4.0
Health insurance									
Medical insurance for urban workers	18 479	53.4	22.9	4230	52.7	54.3	2295	53.9	5.6
Medical insurance for urban residents	8392	24.3	22.8	1916	23.9	54.0	1035	24.3	5.8
Free medical service	1530	4.4	31.4	481	6.0	53.4	257	6.0	4.7
Other	6205	17.9	22.5	1399	17.4	47.7	668	15.7	4.6
Result of preliminary screening									
Only HRFQ‐positive	17 810	51.5	18.3	3252	40.5	44.8	1456	34.2	1.0
Only iFOBT‐positive	14 004	40.5	26.4	3703	46.1	58.3	2158	50.7	7.6
Both positive	2792	8.1	38.4	1071	13.3	59.9	641	15.1	8.1
*District‐level variables*									
Urban‐rural location									
Urban area	22 536	65.1	26.8	6035	75.2	51.5	3110	73.1	5.2
Rural area	12 070	34.9	16.5	1991	24.8	57.5	1145	26.9	6.1
Discount on the cost of colonoscopy									
No	11 561	33.4	16.4	1891	23.6	60.8	1150	27.0	5.3
100‐300 (RMB)	12 188	35.2	19.4	2360	29.4	56.1	1324	31.1	7.3
Free colonoscopy	10 857	31.4	34.8	3775	47.0	47.2	1781	41.9	4.1
*Spatial variables*									
Travel time (min)									
~10	4683	13.5	27.1	1270	15.8	48.0	610	14.3	3.8
~30	20 765	60.0	23.7	4915	61.2	53.3	2622	61.6	5.4
~60	6633	19.2	20.4	1353	16.9	56.5	762	17.9	5.7
>60	2525	7.3	19.3	488	6.1	53.5	261	6.1	8.4
PPR (per thousand people)									
≤50	20 744	59.9	17.6	3653	45.5	58.2	2125	49.9	6.6
>50	13 862	40.1	31.5	4373	54.5	48.7	2130	50.1	4.3
Spatial accessibility (mean ± SD)	0.04 ± 0.04		NA	0.04 ± 0.04		NA	0.04 ± 0.04		NA

Abbreviation: NA, not applicable.

Similar approaches were applied using logistic regression models to predict intestinal lesions and CRC incidence. The random‐intercept logistic models with individuals (level 1) nested in districts (level 2) were used and odds ratio (OR) was estimated.

We used R (version 3.4.4) to conduct the E2SFCA, interpolate the spatial data, generate the maps, as well as perform Cox regression and logistic regression models. Estimates with *P*‐values smaller than .05 were considered statistically significant.

## 3 RESULTS

### 3.1 Participants’ characteristics

There were totally 34 606 people identified with positive preliminary screening results, with an average age of 63.8 years. Most participants were female (60.8%), married (90.0%), received a secondary school education or above (69.1%), and lived in urban areas (65.1%). We observed that 53.4% had the urban employee medical insurance; 8.1% were positive for both HRFQ and iFOBT; and 31.4% had access to the free colonoscopy (Table 1). Additionally, we found that 8026 (23.2%) people underwent colonoscopy, among which 4255 (53.0%) were identified with intestinal lesions. Moreover, among the people with intestinal lesions, 5.4% had been diagnosed as CRC. Based on our estimates, patients with intestinal lesions had similar characteristics with those identified with positive preliminary screening results.

### 3.2 Spatial accessibility of colonoscopy hospitals

**FIGURE 2:**
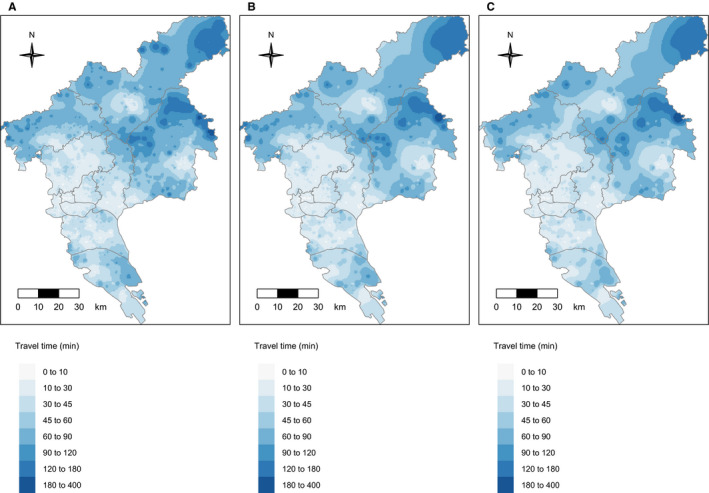
The shortest travel time to colonoscopy hospitals for: A, People with positive preliminary results; B, People receiving colonoscopy exam; C, people with intestinal lesions

We found that 73.5% people identified with positive preliminary screening results could access to the nearest colonoscopy hospital by public transportation within 30 minutes. Among the rest participants, 7.3% needed to travel longer than 60 minutes, and 0.7% more than 120 minutes. Figure 2 shows the spatial variation in travel time to the nearest colonoscopy hospital. The areas with travel time more than 120 minutes were mainly located in the northeast Guangzhou (including Conghua and Zengcheng).

**FIGURE 3:**
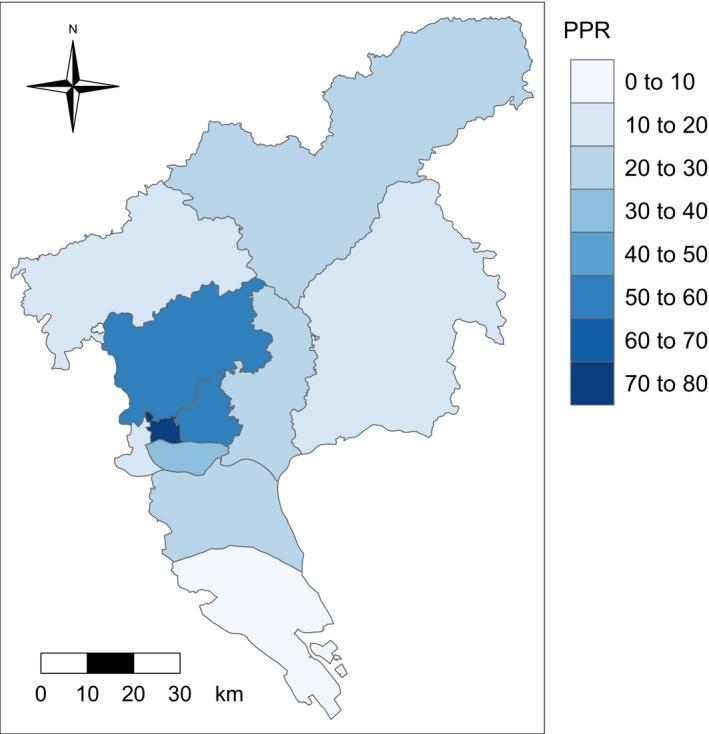
District based physician‐to‐population ratio in Guangzhou

Figure 3 shows the PPR of 11 districts in Guangzhou. We observed the highest PPR (74.8 per thousand people with positive preliminary screening results) in Yuexiu County and the lowest PPR in Nansha County (6.2 per thousand people).

**FIGURE 4:**
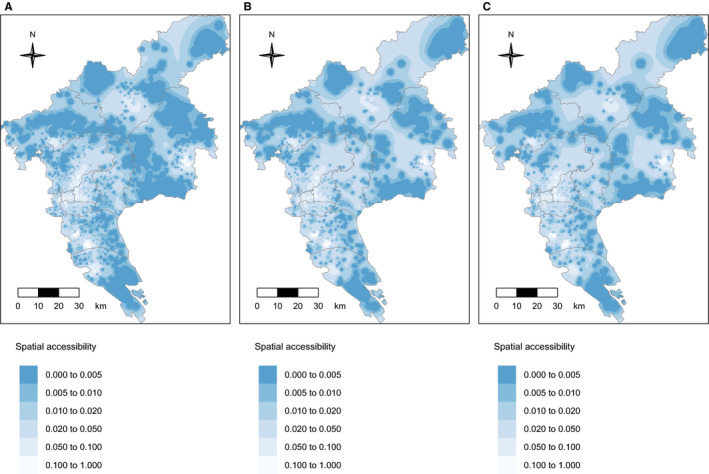
Spatial accessibility to colonoscopy hospitals for: A, People with positive preliminary results; B, People receiving colonoscopy exam; C, people with intestinal lesions

Figure 4 illustrates the spatial accessibility by E2SFCA to gastroenterology hospitals. The spatial variation in E2SFCA scores among the people with preliminary screening positives, the people receiving colonoscopy exam and those with intestinal lesions was similar. We observed that participants in the central of the city (Yuexiu, Tianhe, Haizhu) generally had higher score than those living elsewhere (especially those in the northeast counties such as Conghua and Zengcheng). We found 2695 (7.8%) of participants with positive preliminary screening results had scores higher than 0.1, and 3095 (8.9%) had scores of 0. The estimates were 780 (9.7%) and 566 (7.1%) for the people receiving colonoscopy exams, and 379 (8.9%) and 309 (7.3%) among those with intestinal lesions.

### 3.3 The association between the spatial accessibility and the colonoscopy adherence/ adverse intestinal outcomes

The forward stepwise multivariate Cox regression model in the first step identified eight potential background predictors of colonoscopy adherence: age, gender, education level, occupation, current marital status, preliminary results, discount on the cost of colonoscopy, and rural‐urban location. The model in the second step adjusting for potential individual level confounders found that PPR more than 50 (reference group: ≤50; >50: HR = 1.88, 95% CI: 1.79‐1.97) and higher scores of E2SFCA (HR = 3.78, 95% CI: 2.07‐6.92) were associated with increased adherence. However, in the model 2 adjusting for both individual and district‐level background confounders, no spatial variables were found to be linked to colonoscopy adherence (Table 2).

**TABLE 2:** The association between spatial factors and colonoscopy adherence by multivariate Cox proportional hazard model

	HR_u_ (95% CI)	Model 1^†^	Model 2^‡^
		HR_a_ (95% CI)	HR_a_ (95% CI)
Travel time (min)			
~10	1.00	1.00	1.00
~30	**0.84 (0.79, 0.90)** ***	0.95 (0.89, 1.01)^+^	0.95 (0.89, 1.01)
~60	**0.71 (0.66, 0.77)** ***	0.93 (0.84, 1.02)	1.01 (0.91, 1.11)
>60	**0.67 (0.61, 0.75)** ***	1.12 (0.98, 1.27)^+^	1.03 (0.89, 1.19)
PPR (per thousand people)			
≤50	1.00	1.00	1.00
>50	**1.97 (1.89, 2.06)** ***	**1.88 (1.79, 1.97)** ***	1.10 (0.94, 1.29)
Spatial accessibility	**20.82 (13.21, 32.81)** ***	**3.78 (2.07, 6.92)** ***	1.39 (0.72, 2.74)
Age (y)			
50‐54		1.00	1.00
55‐59		**0.92 (0.84, 1.00)** *	**0.91 (0.83, 0.99)** *
60‐64		**0.84 (0.77, 0.90)** ***	**0.83 (0.77, 0.90)** ***
65‐74		**0.73 (0.68, 0.79)** ***	**0.73 (0.68, 0.78)** ***
Gender			
Male		1.00	1.00
Female		**0.90 (0.86, 0.95)** ***	**0.89 (0.85, 0.94)** ***
Educational level			
Primary school or lower		1.00	1.00
Secondary school		**1.16 (1.10, 1.24)** ***	**1.11 (1.05, 1.18)** **
College or higher		**1.30 (1.20, 1.41)** ***	**1.21 (1.12, 1.32)** ***
Occupation			
Government or public institution		1.00	1.00
Enterprise		**1.12 (1.04, 1.21)** **	**1.15 (1.06, 1.24)** **
Peasant		0.98 (0.90, 1.08)	**1.11 (1.02, 1.22)** *
Unemployed		1.02 (0.94, 1.10)	1.06 (0.97, 1.15)
Other		1.04 (0.99, 1.10)	**1.06 (1.00, 1.12)** *
Marital status			
Married		1.00	1.00
Other		**0.87 (0.80, 0.94)** ***	**0.86 (0.79, 0.93)** **
Result of preliminary screening			
Only HRFQ‐positive		1.00	1.00
Only iFOBT‐positive		**1.67 (1.59, 1.76)** ***	**1.76 (1.67, 1.84)** ***
Both positive		**2.44 (2.28, 2.62)** ***	**2.51 (2.34, 2.69)** ***
Urban‐rural location			
Urban area			1.00
Rural area			0.89 (0.79, 1.00)^+^
Discount on the cost of colonoscopy			
No			1.00
100‐300 (RMB)			**1.19 (1.07, 1.33)** *
Free colonoscopy			**2.12 (1.91, 2.36)** ***

Note

ORs and 95% CIs with *P* < .05 were in bold.

HR_u_: hazard ratios of univariate cox regression models.

HR_a_: hazard ratios of cox regression models adjusting for potential confounder.

^+^ *P* < .10;

* *P* < .05;

** *P* < .01;

*** *P* < .001.

^†^ Adjusted for statistically significant individual‐level variables.

^‡^ Adjusted for statistically significant individual‐ and district‐level variables.

For intestinal lesions incidence, the forward stepwise multivariate logistic regression model in the first step identified seven potential background predictors: age, gender, occupation, preliminary results, health insurance, discount on the cost of colonoscopy, and rural‐urban location. In the final multilevel logistic model adjusting for both individual and district‐level background confounders, people with PPR more than 50 (reference group: ≤50; >50: OR = 0.49, 95% CI: 0.24‐0.99) and higher scores of E2SFCA (OR = 0.20, 95% CI: 0.05‐0.82) were less likely to present with intestinal lesions (Table 3).

**TABLE 3:** The association between spatial factors and intestinal lesions by multivariate logistic model

	OR_u_ (95% CI)	Model 1^†^	Model 2^‡^
		OR_a_ (95% CI)	OR_a_ (95% CI)
Travel time (min)			
~10	1.00	1.00	1.00
~30	**1.24 (1.09, 1.40)** **	1.01 (0.88, 1.16)	1.06 (0.92, 1.21)
~60	**1.40 (1.20, 1.63)** ***	0.96 (0.79, 1.16)	1.00 (0.81, 1.23)
>60	**1.24 (1.01, 1.53)** *	**0.74 (0.57, 0.95)** *	0.99 (0.74, 1.34)
PPR (per thousand people)			
≤50	1.00	1.00	1.00
>50	**0.68 (0.62, 0.75)** ***	**0.74 (0.67, 0.82)** **	**0.49 (0.24, 0.99)** *
Spatial accessibility	**0.04 (0.01, 0.10)** ***	**0.12 (0.03, 0.44)** *	**0.20 (0.05, 0.82)** *
Age (y)			
50‐54		1.00	1.00
55‐59		**1.19 (1.00, 1.42)** *	**1.22 (1.03, 1.45)** *
60‐64		**1.28 (1.09, 1.50)** *	**1.30 (1.11, 1.53)** *
65‐74		**1.41 (1.22, 1.64)** ***	**1.46 (1.26, 1.70)** ***
Gender			
Male		1.00	1.00
Female		**0.55 (0.50, 0.60)** ***	**0.56 (0.51, 0.61)** ***
Marital status			
Married		1.00	1.00
Other		1.14 (0.97, 1.35)	1.08 (0.91, 1.28)
Health insurance			
Medical insurance for urban workers		1.00	1.00
Medical insurance for urban residents		0.97 (0.86, 1.09)	0.95 (0.84, 1.08)
Free medical service		1.05 (0.86, 1.28)	1.19 (0.97, 1.47)^+^
Other		**0.79 (0.69, 0.90)** **	**0.77 (0.67, 0.88)** **
Result of preliminary screening			
Only HRFQ‐positive		1.00	1.00
Only iFOBT‐positive		**1.63 (1.48, 1.80)** ***	**1.66 (1.50, 1.84)** ***
Both positive		**1.75 (1.51, 2.02)** ***	**1.80 (1.56, 2.08)** ***
Urban‐rural location			
Urban area			1.00
Rural area			0.64 (0.40, 1.02)^+^
Discount on the cost of colonoscopy			
No			1.00
100‐300 (RMB)			**0.66 (0.43, 0.99)** *
Free colonoscopy			0.76 (0.44, 1.32)

Note

ORs and 95% CIs with *P* < .05 were in bold.

OR_u_: odds ratio of univariate logistic regression models

OR_a_: odds ratio of logistic regression models adjusting for potential confounder.

^+^ *P* < .10;

* *P* < .05;

** *P* < .01;

*** *P* < .001.

^†^ Adjusted for statistically significant individual‐level variables.

^‡^ Adjusted for statistically significant individual‐ and district‐level variables.

In the case of CRC, the final logistic model adjusting for both individual (Age, Gender, Occupation and Result of preliminary screening) and district‐level (Discount on the cost of colonoscopy) background confounders indicated that no spatial variables were significantly associated with CRC incidence (Table 4).

**TABLE 4:** The association between spatial factors and colorectal cancer incidence by multivariate logistic model

	OR_u_ (95% CI)	Model 1^†^	Model 2^‡^
		OR_a_ (95% CI)	OR_a_ (95% CI)
Travel time (min)			
~10	1.00	1.00	1.00
~30	1.55 (0.98, 2.45)^+^	1.34 (0.82, 2.18)	1.34 (0.82, 2.18)
~60	1.60 (0.95, 2.70)^+^	1.44 (0.77, 2.71)	1.52 (0.80, 2.88)
>60	**2.46 (1.34, 4.53)** **	2.02 (0.94, 4.36)^+^	2.02 (0.94, 4.37)^+^
PPR (per thousand people)			
≤50	1.00	1.00	1.00
>50	**0.63 (0.48, 0.83)** **	0.78 (0.58, 1.05)	0.86 (0.48, 1.53)
Spatial accessibility	0.06 (0.00, 1.96)	3.68 (0.05, 26.72)	3.55 (0.04, 287.14)
Age (y)			
50‐54		1.00	1.00
55‐59		1.51 (0.77, 2.96)	1.50 (0.76, 2.94)
60‐64		1.23 (0.65, 2.34)	1.22 (0.64, 2.32)
65‐74		**1.90 (1.06, 3.42)** *	**1.89 (1.05, 3.39)** *
Gender			
Male		1.00	1.00
Female		**0.70 (0.53, 0.92)** *	**0.69 (0.53, 0.91)** *
Occupation			
Government or public institution		1.00	1.00
Enterprise		**0.57 (0.33, 0.99)** *	**0.57 (0.33, 1.00)** *
Peasant		0.70 (0.42, 1.15)	0.70 (0.42, 1.17)
Unemployed		0.86 (0.52, 1.41)	0.87 (0.53, 1.43)
Other		1.17 (0.84, 1.64)	1.18 (0.84, 1.65)
Result of preliminary screening			
Only HRFQ‐positive		1.00	1.00
Only iFOBT‐positive		**7.41 (4.33, 12.68)** ***	**7.44 (4.34, 12.75)** ***
Both positive		**8.41 (4.68, 15.11)** ***	**8.38 (4.66, 15.06)** ***
Discount on the cost of colonoscopy			
No			1.00
100‐300 (RMB)			1.32 (0.90, 1.93)
Free colonoscopy			1.13 (0.64, 1.99)

Note

ORs and 95% CIs with *P* < .05 were in bold.

OR_u_: odds ratio of univariate logistic regression models

OR_a_: odds ratio of logistic regression models adjusting for potential confounder.

^+^ *P* < .10;

* *P* < .05;

** *P* < .01;

*** *P* < .001.

^†^ Adjusted for statistically significant individual‐level variables.

^‡^ Adjusted for statistically significant individual‐ and district‐level variables.

## 4 DISCUSSION

In this study, 23.2% people with positive preliminary screening results underwent colonoscopy in Guangzhou during follow‐up period which was lower than the percentages for other parts of China.^11^, ^12^ Our results also suggested that colonoscopy has detected 53.0% residents' intestinal lesions, which could help these people achieve early detection and treatment, so as to timely control the lesions. We also assessed the spatial variation in the accessibility to colonoscopy hospitals in Guangzhou. In addition, we found that higher PPR and E2SFCA scores were associated with significantly lower intestinal lesions incidence.

We found that the central areas of Guangzhou (Yuexiu, Tianhe) were characterized with higher E2SFCA scores, higher PPR and less travel time as compared with other areas of Guangzhou, particularly the northeast (Conghua, Zengcheng). We found that a significant proportion of residents in Guangzhou were not able to reach any colonoscopy hospital within 60 minutes, the majority of which lived in rural areas. However, we also found population with high spatial accessibility (had an E2SFCA score of 0.05 or higher) and less travel time (arrived at a nearest colonoscopy hospital within 30 minutes) in rural areas. A potential interpretation was that medical resources were unevenly allocated and were less accessible for those living far away. Inconvenient transportation also played an important role. The accessibility of public transportation in rural areas is far less than that in urban areas.

We found that the lowest PPR among the 11 districts of Guangzhou was 6.3 per thousand people, and the medical resources in rural areas of Guangzhou or even the entire region were far from meeting the needs of colonoscopy. In addition, in the current study, we only included participants with positive preliminary screening findings identified between 2015 and 2016. As the CRC screening program continues, we may expect a growing number of preliminary screening positives being discovered. Therefore, it is important and urgent to increase the coverage of colonoscopy.

We did not find any spatial factors significantly associated with the average colonoscopy adherence among residents with positive preliminary screening findings when both individual and district‐level variables were controlled. Based on our estimates, the effects of spatial factors on the average colonoscopy adherence were mainly due to differences in demographic characteristics (age, gender, etc) of the residents, and discount on the cost of colonoscopy of the location where they lived. Other studies also reported similar conclusions.^25^, ^41^, ^42^ Our results show that the increase in discount would promote colonoscopy adherence. However, residents in urban areas generally would get more discount than rural residents, which was an important spatial inequality in medical resource in China.

After controlling both the individual and district characteristics, we found that intestinal lesions incidence could be attributed to spatial accessibility. Population in districts with PPR more than 50 tended to have a lower rate of intestinal lesions, while those from the areas with decreased E2SFCA scores tended to have higher rate of intestinal lesions. However, travel time was not associated with intestinal lesions. Therefore, the spatial accessibility may affect intestinal lesions incidence mainly through spatial inequality in medical resources. The reason for the above results may be as following: Lack of medical resources in these areas might be associated with the lack of colonoscopy appointment, resulting in a potential underutilization of health screening services. In addition, greater number of hospitals concentrated in urban regions and associated higher healthcare service uses might lead to lower rate of intestinal lesions. We also controlled the discount on the cost of colonoscopy, as it was the most important indicator of healthcare service utilization, directly affecting the colonoscopy exam adherence.

We did not find any spatial factors significant for CRC. A potential interpretation was the sample size of cancer patients is small and the statistical power was limited. Although we observed similar results for intestinal lesions in the univariate analysis, future studies with a larger sample size will be needed to confirm our findings.

Policies against the spatial inequality in medical resources will be important. First, CRC screening programs should be promoted in rural areas, particularly those with low spatial accessibility. Specifically, efforts should be put in expanding the colonoscopy coverage in rural areas. However, since medical resources are usually limited, it is critical to develop an efficient case management model. The Optimization of Sequential Screening Scheme currently being used in China could effectively alleviate the burden of colonoscopy as it excludes a large number of people with low risk of CRC.^43^ Our results suggest that this scheme needs to be further optimized, including simplifying the colonoscopy appointment process, opening outpatient clinics that serve the CRC screening program, etc

This study has several limitations. First, we only included residents with positive screening findings in 2015‐2016. The spatial accessibility, travel time, and PPR estimates based on these participants may not fully represent the situation among the general population in Guangzhou. Second, accessibility and travel time were calculated based on public transportation. We did not consider other forms of transportation such as personal vehicle. Third, we were not able to control income, a potential confounder on the association between the accessibility and adherence, in our analysis since this information was not collected by the screening system. Nevertheless, the impact of income could to some extent be captured by the impact of variables such as health insurance and occupation in our models. Forth, the health insurance usually covered inpatients but not outpatients, however, the inpatient/outpatient identification information was not collected by the screening system, thus, we did not distinguish these two types of patients.

In summary, we found significant inequality of spatial accessibility in Guangzhou, with the central areas reported with higher E2SFCA scores, higher PPR, and less travel time. We also identified potential associations between the increased incidence of intestinal lesions and spatial inequalities of medical resources. Population in districts with PPR more than 50 per thousand people tended to have a lower rate of intestinal lesions. Moreover, decreased E2SFCA scores was associated with increased incidence of intestinal lesions. Policies against the spatial inequality were needed.

## CONFLICT OF INTEREST

The authors declare no conflict of interest.

## Data Availability

Research data are not shared due to privacy and ethical restrictions.
